# The (In)Dispensability of Environmental Justice Communities: A Case Study of Climate Adaptation Injustices in Coastal Louisiana and Narratives of Resistance

**DOI:** 10.1089/env.2021.0098

**Published:** 2022-08-09

**Authors:** Simone Justine Domingue

**Affiliations:** Dr. Simone Justine Domingue is postdoctoral research fellow at the Department of Geography and Environmental Sustainability, University of Oklahoma, Norman, Oklahoma, USA.

**Keywords:** critical environmental justice, climate change, coastal hazards, adaptation planning, Louisiana

## Abstract

This case study analyzes how climate adaptation actors in coastal Louisiana undermine the justice concerns of coastal communities comprising Native American, Black, Southeast Asian, Hispanic, and working-class people. The homes, livelihoods, and cultures of these environmental justice (EJ) communities are threatened not only by climate disasters and ecological degradation, but also by adaptation projects proposed and backed by the state and federal governments and restoration nonprofit organizations. Drawing on 74 in-depth interviews, I analyze discourses from adaptation actors (government staff, scientists, engineers, and restoration advocates) and from coastal community leaders. Findings from the case study reveal how climate adaptation actors reference a socially constructed “bigger picture” to justify negative externalities of coastal projects while also undermining community concerns regarding their own survival. Findings also show how members of coastal communities discuss their survival, resist harmful narratives, and assert their indispensability. I conclude by connecting these themes to critical EJ research, particularly the racist underpinnings of utilitarian environmental decision making. This case study demonstrates the need to examine institutional actors' resistance to integrating justice into climate adaptation planning and action.

## Introduction

Because the planet is on a trajectory of increased warming due to unchecked greenhouse gas emissions, more cities and states are implementing measures to adapt to sea level rise and severe weather events. Emerging research on climate adaptation injustice indicates that these activities create winners and losers, producing and reinforcing existing inequalities for environmental justice (EJ) communities (or people of color and working-class communities overburdened by environmental ills and hazards).^1^^,^^2^^,^^3^^,^^4^^,^^5^^,^^6^

Coastal Louisiana is a case in point. The area is often referenced as *the* harbinger of climate catastrophe for coastal states in the United States.^7^

The state has been struck by two consecutive seasons of record breaking hurricanes, including Hurricane Ida that made landfall on August 29th, 2021, ripping through the homelands of marginalized bayou people. Although their vulnerability is undeniable, there have been few sustained efforts on the part of dominant institutions to help coastal communities adapt to climate change, and these communities are suffering injustices as a result.^8^ EJ communities are left filling in the gaps, often through mutual aid networks.^9^ In the case of southwestern Louisiana, EJ communities in Lake Chalres are still struggling to find housing after Hurricane Laura and find resources to make resilient housing repairs.^10^

This case study of coastal adaptation actors in Louisiana illustrates how these climate injustices persist. This case study connects to critical EJ perspectives and confronts the ideological milieu in which climate adaptation actors are embedded, while also recognizing how EJ community leaders challenge dominant narratives contributing to their marginalization vis-à-vis privileged communities. A critical approach to EJ scholarship highlights state violence and the “expandability” of EJ communities, meaning how they are treated as disposable by institutions.^11^^,^^12^

This disposability is undergirded by the ideological assumption that the well-being and fate of EJ communities—particularly people of color—are delinked from that of the larger (white) society. This case study elucidates how this happens on the ground, analyzing how adaptation actors in coastal Louisiana do recognize some forms of social vulnerability yet still frame the priorities of EJ communities as different than, and opposed to, a socially constructed idea of the common good.

## Problem

The slow-moving environmental disaster of coastal land loss is currently threatening the social and ecological landscape of coastal Louisiana. The disaster is inextricably tied to anthropogenic climate change. The warming of the planet causes sea level rise and strengthens tropical storms and hurricanes, factors that further erode already-vanishing coastal wetlands.^13^^,^^14^ For coastal villages, towns, and cities, the loss of land means the deterioration of fish and wildlife habitat as well as the loss of an important first line of defense against hurricanes. The disaster is not going unnoticed in Louisiana. The state government, in partnership with private and civil sectors, has launched an ambitious response, culminating in a multibillion dollar plan called Louisiana's Comprehensive Master Plan for a Sustainable Coast.

The compound disaster of land loss and climate change is yet another crisis for racially marginalized and economically dispossessed communities facing the convergence of life-threatening hurricanes, pandemic, racism, and toxic contamination.^15^^,^^16^^,^^17^ These communities comprise Native American, Black, Southeast Asian, Hispanic, and working-class Cajun and Creole people who came to reside in coastal regions as a result of colonialism, slavery, and imperialism.^18^^,^^19^^,^^20^

For example, citizens of the Pointe-au-Chien Indian Tribe, Grand Caillou/Dulac band of Biloxi-Chitimacha-Choctaw, United Houma Nation, Isle de Jean Charles band of Biloxi-Chitimacha-Choctaw, and the Grand Bayou Atakapa-Ishak/Chawasha reside in the bayous of southeastern Louisiana and trace their ties to this land back to the early 19th century when they were violently pushed south by European settlers. Displacement of these peoples threatens their survival because their livelihoods are tied to the coast, they rely on social capital embedded in community to withstand economic and environmental disasters, and because having a homeland helps pass on cultural traditions.

Specific measures (such as resettlement practices and the building of engineered structures) produce harms for EJ communities.^21^^,^^22^^,^^23^ Massive infrastructure projects (such as the Mid-Barataria and Mid-Breton sediment diversions) included in the state's Coastal Master Plan are opposed by downstream fishing communities, such as those in lower Plaquemines Parish, home to Vietnamese and Cambodian fisher folk. They fear such projects will destroy their way of life. This is because projects will alter natural resources (fish, wildlife, and vegetation). Projects will also cause more flooding in downstream areas, such as the traditional homelands of the Grand Bayou Atakapa-Ishak/Chawasha tribe.^24^

The state agency in charge of implementing the state's Coastal Master Plan, the Coastal Protection and Restoration Authority, acknowledges that the plan will not protect all coastal inhabitants and offers no concrete options for community adaptation or cultural preservation, implying that less-densely populated coastal communities will have to relocate, although individuals, families, and communities are constrained in their options for doing so. The lack of investment in these coastal communities makes these places precarious, meaning many are forced to leave or are displaced when a disaster comes.

Hurricane Ida provides a recent example. Initial reports after the storm showed that tribal villages experienced catastrophic and complete losses, having housing units flattened, flooded, or blown away.^25^ Although residents evacuated out of harms way, it is uncertain as to how many will be able to return and rebuild. Another issue is how governments prioritize flood protection infrastructure. For example, a historic Black community in Ironton was excluded from the building of new levee systems and as consequence received devasting flooding from Hurricane Ida—flooding so powerful that it scattered caskets from underground cemeteries all over the town.^26^

Injustices are not inevitable; the state could do more to actively prioritize the needs of EJ communities while addressing root causes of the disaster (such as compelling oil and gas companies to provide compensation for damages and remediate wetlands). Drawing from cultural discourses of climate adaptation actors (government staff, scientists, engineers, restoration professionals and advocates, etc.), this case study provides novel insights into why it is the case that injustices persist.

## Theoretical Framework: Climate Adaptation Injustice and Critical EJ

There are many explanations for how climate-related plans and projects create injustices. Hazard mitigation plans often emphasize personal preparedness for severe weather events while ignoring structural inequalities that create hazardous conditions and exposures for marginalized people.^27^^,^^28^^,^^29^^,^^30^ For example, people of color are more likely to not have adequate cooling and heating in places where they live and work, reside in low-lying elevations or floodplains, live and work in communities with failing infrastructure, or live next to polluting industries and toxic hazard sites—conditions linked to structural racism.^31^^,^^32^^,^^33^

Meanwhile, scholars have shown that communities of color and low-income communities are more likely to be forced to relocate because flood protection infrastructure is not prioritized in the places they live.^34^ People of color and low-income people are less likely to benefit from disaster relief and home buyout programs.^35^^,^^36^^,^^37^ In addition, in terms of emissions reductions, Indigenous people and less-affluent nations are at jeopardy of losing land rights with the uptick in renewable energy and carbon-offsetting projects.^38^^,^^39^^,^^40^

Anguelovski *et al*. argue climate adaptation plans produce these types of environmental injustices because of two processes: acts of omission and acts of commission. Acts of omission occur when planning bodies disregard the adaptation needs of vulnerable or historically marginalized groups.^41^ Acts of commission take place when planning bodies actively prioritize stakeholders who already have resources and privilege in a community. However, the claims of marginalized people can be discredited even when they are included, and this is particularly true for communities of color.

For example, critical EJ scholars demonstrate how proenvironmental and colorblind discourses erase racial inequalities and allow government staff, planners, and citizens to resist antiracist policies and practices.^42^^,^^43^^,^^44^ In addition, institutions privilege scientific knowledge over local, indigenous, or experiential knowledge.^45^ Government and nonprofit actors undermine community claims by asserting their scientific authority or framing themselves as responsible practitioners, acting in accordance with their organizational mission or mandate.^46^^,^^47^

Scholars note that this is enacted through “boundary work,” but gaps remain in understanding how actors discursively justify their positions, especially when underlying vulnerabilities or inequalities are not necessarily denied. This case study builds on such scholarship and adds to it by revealing the discourses used by adaptation actors to dismiss community justice concerns and justify externalities that they may suffer, while also highlighting narratives of resistance from EJ community leaders.

## Research Design

### Case selection

The case of adaptation planning in coastal Louisiana is illustrative because this area has a legacy of both environmental injustices and strong grassroots environmental activism. The oil and gas industry has left an indelible mark on Louisiana, directly shaping its culture and politics while degrading its environment and diminishing the health and quality of life of EJ communities. The name “Cancer Alley” is used to describe the multitude of oil and gas refineries and industrial plants along the Mississippi River Corridor. These sites are located adjacent to majority Black communities where residents are subjected to constant health hazards and threats posed by climate change (and often resist the siting of new facilities).^48^

For these reasons, understanding how climate adaptation happens in this extreme setting could yield particularly salient insights for EJ research. In addition, findings from this case could inform the creation of typologies or matrices of climate adaptation injustices because of these factors: the effects of climate change are pronounced in coastal Louisiana, climate is affecting both rural and urban communities, and because adaptation is happening under considerable institutional, financial, and political constraints.

### Sampling

A main source of data for this case study is in-depth interviews. I drew findings for this case study from interviews with 74 people. This case study was a part of a larger project on coastal land loss in Louisiana. The sampling strategy was purposeful. I recruited individuals who worked on projects for long periods of time and who had very specialized knowledge about projects. I also sought out individuals who were deeply embedded in communities. Data collection and interviews (both in-person and remote) happened over the summers of 2018 and 2019 and in the year 2020.

Interviewees included individuals who worked in or with coastal and community organizations identified through a review of scholarship on coastal land loss and through an analysis of texts (newspaper articles, press releases, blogs, etc.) about coastal risks and coastal communities.

Interviews included coastal scientists and social scientists working at Universities or research institutes on land loss-related issues and restoration projects (13); engineers, planners, and project managers working on computer modeling of the coast, designing river diversions, and implementing the state's master plan (13); state and federal government administrators coordinating coastal funding and public engagement programs (7); coastal nonprofit employees (26); and community leaders and organizers (15).

In this sample of interviewees, I identified 44 as women and 30 as men. I identified 60 as white or white and Cajun, 8 identified as Black or Black and Creole, 3 as Vietnamese American or South Asian, and 3 people identified as Native American or Native American and Creole. The sample of community members, in particular, is not meant to be exhaustive but rather provides empirical evidence for how community members respond to coastal projects and planning.

I used a semistructured interview guide for data collection. However, I was flexible about how I asked questions, let the interviewee direct the flow of the interview, and used probing questions to get participants to elaborate on topics in their own words. This strategy is useful for getting rich data from interviews. I used a process of both open and theoretically oriented coding in NVivo to create and refine themes from interview texts. I used pseudonyms to keep the identities of individuals I interviewed confidential, and all research was approved by the University of Colorado's Institutional Review Board.

## Results

The following sections describe two major findings from the case study. First, I show how discourses frame the concerns of frontline communities as standing in the way of what climate adaptation actors see as Louisiana's only option for dealing with land loss and climate change—building large-scale flood protection and coastal restoration projects. I show how this process involves the social construction of a “bigger picture,” or shared common good, that excludes frontline communities. I then show evidence for how community viewpoints are described by these actors and contrast them to narratives of community members and advocates.

### “The bigger picture”: social constructions of the common good

Individuals working on large-scale climate adaptation projects discussed how they know that Louisiana cannot be “saved” in the sense that the whole coastline cannot be put back to the way it was historically. They admit that land is never going to be rebuilt in some areas and that there is going to be more loss of land in the future. However, they were not deterred by this, saying that it was their mission to “preserve what we can” or do the “most we can” with what we have (referring to river sediment and funding). Part of this hopeful, yet pragmatic, take was a commitment to doing “the most good,” meaning building projects that maximize land building and flood protection potential.

This is cast as an obvious way of prioritizing resources; large-scale engineered projects, that is, river diversions, are regarded as the *best* projects. This is because they are predicted by the state's computer models to build the most land for the least amount of money over time, benefitting numerous people in urban centers. However, these projects will alter the ecology of estuaries and increase flooding risk in some downstream areas, leading to significant problems for people dependent on those resources and vulnerable to flood risk.

When weighing the notion that projects might be harmful to downstream coastal communities, such as the tribal village in Grand Bayou and Southeast Asian and working-class fishing villages, scientists, engineers, and advocates of the diversions invoked the language of a “bigger picture” to justify any negative externalities. For instance, Rebecca, who works for the state, said this in our interview: “We understand the concerns that this project may disrupt people's way of life, but it is for the bigger picture.” Similarly, Mary, who engages faith leaders in prodiversion advocacy as part of a nonprofit said:

There are plenty of people who are anti-diversion who have very legitimate concerns about how diversions will change the estuary, the whole point of them is to change the estuary, and we do have to figure out how to address some of those people's concerns, but there is a bigger picture of what needs to happen in the long term in coastal Louisiana if any of us want to keep living here.

Here, Mary acknowledges the need to address community concerns but does not mention that the diversions were designed without their input, making it is dubious as to whether the state would adequately understand and address their concerns. Henry, who works in state government administration, expressed the idea that it was the state's duty to preserve the “common good,” meaning that the state should not manage the coastal zone according to the interests of one stakeholder, such as fishers. He said that the idea that the state is a protector of a common good is supported by public trust doctrine, saying “who else but the state can have this prerogative?” The idea of the common good meant that the state was going to focus on maintaining as much land area as possible for the benefit of the greatest number of people.

Even though the river diversions were discussed as an absolute imperative, individuals working on this project and other related restoration and flood protection projects conceded that they can only preserve and protect limited amounts of land into the future. They did not discuss prioritizing resources for adaptation based on what areas of coastal land are the most culturally or ecologically significant to communities who are the most reliant on those resources, nor were projects intended to help these communities adapt or transition to new livelihoods. Most people I interviewed emphasized the need to focus on projects that have “the most bang for your buck,” even though they understood that gains from large projects were not going to preserve the coastline in a long-term sense.

### “Delusional” people: mischaracterization of community viewpoints and counter narratives

I heard narratives describing large-scale restoration projects as the best option because it meant “controlled” changes to the coastline. For instance, when discussing opposition to river diversions, Monique argued that fisheries would be destroyed even if the diversions were not built. She said this regarding fishing communities:

This is their livelihood, this is all that they have known, yet they see all of the changes, and this is change without projects in place.

She continued, stating that there are only two options for the coast, build river diversions or take no action:

We call it a future without action, and that means that you do nothing. And without action the coast will no longer exist, there will be no more fisheries. There will be a total collapse, right? Or you have this future with action, and it does mean changes, but you know, we feel like it means controlled changes.

This statement conveys the predication that fisheries will collapse in the future if there are no diversions but fails to recognize the concerns or desires of community members who want to adapt, have few resources to do so, and will be immediately negatively affected by diversions.

Christina, who works in coastal policy also expressed the sentiment that some individuals are “delusional” for thinking that they can continue to live in coastal areas, not understanding that their homes are not economically justifiable to “save.” She said frankly:

Some places will be saved, and other places won't be saved. Um, so I think that is a really tough pill to swallow for people. You know, you live in the bayou, you are not going to get saved. If you live in New Orleans, you might be saved. Right? There is just certain places that are economically justifiable to put the money into, and other ones that aren't, and I don't think that people really realize that here. Or they are just kind of delusional, living way out in the bayou—no one is ever going to build a levee for you.

She is referring specifically here to the way the Army Corps of Engineers and other government entities conduct cost–benefit analyses for the building of levees and flood protection structures. These analyses inherently disadvantage poorer communities with lower property values. Indeed, EJ communities in the lower reaches of the coast were excluded from new and improved ACOE levee systems built after Hurricane Katrina (a factor that led to significant flooding during Hurricane Ida^49^).

The types of narratives mentioned by adaptation actors do not capture the range of concerns and sentiments of frontline community members. Community members I spoke to do understand the fleeting and precarious nature of the coast. Daniel, a scientist who works with coastal residents, noted that fisher folk are not opposed to “having to give up something”; they just do not want to “give up everything,” meaning that they are willing to adapt in some ways, but they are concerned that their lives will be upended by the way the state prioritizes resources for adaptation.

He added that they are frustrated by how the state makes decisions without involving them and without drawing on their own applied expertise and knowledge about the places where they live and work every day. Kim, who came to Louisiana from Vietnam and now advocates for fishing communities in southeastern Louisiana, explained to me in an interview that her people are “dying for restoration.” She said they want to be included in decisions about diversions and not case aside because they lack formal education.

She also makes the point that the environment is but one of many factors that threaten the survival of her community (along with pandemic, structural racism, and expanding economic inequality) and that contributes to her people's sense of expandability. Explaining that she cannot even grasp what “environmental justice” would mean to her community she said bluntly, “I don't know what that is or what it entails, all I know is there's no justice, at all, environmental wise, economic wise, community wise, culture wise.”

Additional community leaders and members that I spoke to grappled with the finite nature of their community. Bobby, who runs a coastal community organization, described how he accepted that his community cannot stay where it is forever, poignantly saying:

It is about embracing the fact that will not be forever, and there is something beautiful about that. It's like the difference between getting live flowers and plastic flowers, for your birthday or Valentine's. You sort of treasure those flowers and how beautiful and fragile and delicate they are, where the plastic flowers might go in a box and collect dust somewhere and end up at a garage sale. I think [we are] coming to that mindset of cherish [this place] because it is beautiful, and will go away, and celebrate that.

Community advocates believe their ecological knowledge could be useful in designing projects that are still successful in rebuilding wetlands, but that will not negatively affect communities. Donna, a Native American tribal leader in a southeastern Louisiana bayou community, put it like this:

If you can look down the road and see that you've just wiped out an entire community and the people by what you've just proposed to do, then that should not even be an option…it all boils down to love, really. If what you are doing does not have love for everyone—and that is all of our relations, that's the plants, the animals, the people. If you are going to impact those, then you really should not be [doing it]…There are other ways [to restore the coast] based on our own traditional knowledge that could actually work and end up having little to no negative impact whatsoever. And that's definitely because of the way that we function as a people, you bring harm to none.

In this quote, Donna sums up her tribe's philosophy as one of “doing no harm” that is bound up in respect for people and the natural world. She does this to contrast the state's approach, which she says does not adequately recognize their value and does not consider alternative approaches to restoration and adaptation. Tribes support alternative approaches, such as backfilling oil and gas canals, because they address the ecological harms done by oil and gas companies—a topic that the state's adaptation planning skirts around.^50^

## Discussion

The state's climate adaptation plan could severely harm EJ communities, meaning they will be displaced from their homes, lose social capital they depend on, be forced to abandon cultural traditions, and lose their only sources of income or savings. These outcomes are not inevitable; climate adaptation efforts could focus more resources on EJ communities and prioritize their needs. The narratives already described start to explain why this is not the case. One reason is that climate adaptation planning happens in accordance with utilitarian environmental management philosophy.

Utilitarian management of the environment aims to maximize the number of people benefiting from government environmental policies and is associated with the use of cost–benefit analyses in decision making.^51^ This management technique also aligns with neoliberal reforms in government, where the slashing of public spending, emphasis on fiscal austerity, and concern for continued capital accumulation encourage the use of strategies that are deemed economically efficient, meaning we have the greatest economic returns per unit of input.^52^

The findings from this case study also make visible an assumption that is taken for granted in the underpinnings of decision making; the prioritization of the marginalized (particularly people of color) necessarily works against interests of a larger public. This is also consistent with a “zero-sum” logic that undergirds white supremacist ideology—an assumption that a critical approach to EJ counters.^53^^,^^54^^,^^55^

The community narratives highlighted previously display how viewpoints of community leaders and activists invert a zero-sum framing. For example, Donna made the point that her tribe's philosophy of “doing no harm” could be seen as an asset in a collective struggle to address climate change and growing ecological problems. Black climate justice activist and native of coastal Louisiana, Colette Pichon Battle, has made similar points about the indispensability of EJ communities.

In a TED talk she communicated this by stating, “the only way you are going to survive is for us to figure out how to reach a shared liberation together.”^56^ In a speech to coastal restoration professions, she passionately stated that the climate crisis in Louisiana is a direct result of an extractive and oppressive political–economic system, challenging Louisiana's petro-dominated political order as one that benefits the few while creating catastrophe for the many.^57^

## Conclusion

In sum, findings from this case study reveal how the needs of the most marginalized are discursively evaluated within a constraining framework for action, one that pits their needs against those of a socially constructed greater good. These findings connect to a critical EJ perspective. This perspective challenges assumptions undergirding technocratic policy discourse, particularly the taken-for-granted assumption that justice is inherently at odds with climate adaptation goals.

This case study demonstrates the need to develop conceptual frameworks for studying *resistance* to integrating justice into climate adaptation, or how and why concerns over justice become marginalized within specific settings. Future research should explore resistance in terms of other climate-related programs and policies, including federal disaster mitigation and recovery funding. This case study underscores the continued need to partner with community members and grassroots organizations to codesign climate adaptation solutions and further illuminate community viewpoints.

Finally, this case study also offers an important lesson for both researchers and practitioners alike: when grappling with the climate crisis, we should reject narratives that devalue whole communities or that delink their well-being and human rights from that of the broader public. This is particularly important to this current social moment, as action to address climate change is increasingly regarded as urgent and necessary and because these actions are known to disproportionately burden some segments of society while benefitting others.

## Author Disclosure Statement

No competing financial interests exist.

## Funding Information

The research was supported by the National Science Foundation, Award #2001738 and The National Oceanic and Atmospheric Administration Regional Integrated Sciences and Assessments, Award #NA18OAR4310337.

## Notes

^1^
David Ciplet and Jill Harrison. “Transition Tensions: Mapping in Movements for a Just and Sustainable Transition.” *Environmental Politics* 29 (2020): 435–456.

^2^
Breena Holland. “Procedural Justice in Climate: Political Capabilities and Transformation Change.” *Environmental Politics* 26 (2017): 391–412.

^3^
Matthew Jujonas, Erin Seekamp, Louie Rivers III, and Bethany Cutts. “Uncovering Climate Injustice with an Adaptive Capacity Assessment: A Multiple Case Study in Rural Coastal North Carolina.” *Land Use Policy* 94 (2020): 104547. DOI:10.1016/j.landusepol.2020.104547.

^4^
Jesse M. Keenan, Thomas Hill, and Anurag Gumber. “Climate Gentrification: From Theory to Empiricism in Mimi-Dade County Florida.” *Environmental Research Letters* 13 (2018): 054001.

^5^
Elizabeth Marino. “Adaptation Privilege and Voluntary Buyouts: Perspectives on Ethnocentrism in Sea Level Rise Relocation and Retreat Policies in the U.S.” *Global Environmental Change* 49 (2018): 10–13.

^6^
Linda Shi, Eric Chu, Isabelle Anguelovski, Alexander Aylett, Jessica Debats, Kian Goh, Todd Schenk, Karen C. Seto, David Dodman, Debra Roberts, J. Timmon Roberts, and Stacy VanDeever. “Roadmap Towards Justice in Urban Climate Adaptation Research.” *Nature Climate Change* 6 (2016): 131–137.

^7^
Jeffrey Thomas. “New Orleans is the Canary in the Coal Mine for Coastal Cities.” CNN (October 8, 2017) <https://edition.cnn.com/2017/10/08/opinions/new-orleans-lessons-other-hurricane-cities-thomas/index.html>. (Last accessed on September 2, 2021).

^8^
Shirley Laska (ed). *Louisiana's Response to Extreme Weather: A Coastal State's Adaptation Challenges and Success*. (Switzerland, Springer, 2020).

^9^
Philip Kiefer. “’We Can't Depend on the Government’” Mutual aid groups work to fill needs for immigrant communities after Ida.” The Lens (September 28, 2021). <https://thelensnola.org/2021/09/28/we-cant-depend-on-the-government-mutual-aid-groups-work-to-fill-needs-for-immigrant-communities-after-ida/?utm_medium=email>. (Last accessed on October 1, 2021).

^10^
Disaster Justice Network. “Resilient Rebuilding in Lake Charles featured on KPLC News.” (July 12, 2021). <http://disasterjusticenetwork.org/2021/07/resilient-rebuilding-in-lake-charles-featured-on-kplc-news>. (Last accessed on October 1, 2021).

^11^
David Pellow. *What is Critical Environmental Justice?* (Cambridge, Polity Press, 2017).

^12^
Ryan Holifield, Michael Porter, and Gordon Walker. “Introduction Spaces of Environmental Justice: Frameworks for Critical Engagement.” *Antipode* 41 (2009): 591–612.

^13^
Donald Boesch. “Managing Risks in Louisiana's Rapidly Changing Coastal Zone.” Shirley Laska (ed). *Louisiana's Response to Extreme Weather: A Coastal State's Adaptation Challenges and Success*. (Springer, 2020), 35–62.

^14^
John W. Day, Craig Colten, and Paul Kemp. “Mississippi Delta Restoration and Protection: Shifting Baselines, Diminishing Resilience, and Growing Nonsustainability.” Eric Wolanski, John W. Day, Michael Elliott, and Ramesh Ramachandran (eds). C*oasts and Estuaries: The Future*. (Elsevier, 2019), 167–186.

^15^
Ada Yokota. “How Vulnerable is Your Community to Coronavirus? These New Maps Reveal a Familiar Pattern.” Grist (April 2, 2020). <https://grist.org/justice/how-vulnerable-is-your-community-to-coronavirus-these-new-maps-reveal-a-familiar-pattern/>. (Last accessed on September 2, 2021).

^16^
Giles Clarke. “UN Says Environmental Racism in Louisiana's Cancer Alley Must End.” Grist (March 5, 2021). <https://grist.org/justice/united-nations-environmental-racism-cancer-alley-louisiana/>. (Last accessed on September 2, 2021).

^17^
John Schwartz and Hiroko Tabuchi. “When Hurricanes Lead to Industrial Fires, Minority Neighborhoods Can Take a Hit.” The New York Times (August 27, 2020). <https://www.nytimes.com/2020/08/27/climate/hurricane-laura-fire-pollution.html>. (Last accessed on September 2, 2021).

^18^
Carl Brasseaux. “Creoles.” <https://cls.louisiana.edu/node/174>. (Last accessed on February 26, 2021).

^19^
Barry J. Ancelet. “Negotiating the Mainstream: The Creoles and Cajuns in Louisiana.” *The French Review* 80 (2007): 1235–1255.

^20^
Nathan Jessee. “Community Resettlement in Louisiana: Learning from Histories of Horror and Hope.” Shirley Laska (ed). *Louisiana's Response to Extreme Weather: A Coastal State's Adaptation Challenges and Success*. (Springer, 2020), 147–184.

^21^
Craig E. Colten, Jessica R.Z. Simms, Audrey A. Grismore, and Scott A. Hemmerling. “Social Justice and Mobility in Coastal Louisiana, USA.” *Regional Environmental Change* 18 (2018): 371–383.

^22^
Julie Maldonado. *Seeking Justice in an Energy Sacrifice Zone*. (New York, Routledge, 2018).

^23^
Scott A. Hemmerling, Monica Barra, and Rebecca H. Bond. “Adapting to a Smaller Coast: Restoration, and Protection, and Social Justice in Coastal Louisiana.” Shirley Laska (ed). *Louisiana's Response to Extreme Weather: A Coastal State's Adaptation Challenges and Success*. (Springer, 2020), 113–144.

^24^
Barry Yeoman. “As Sea-Level Rise Threatens Their Ancestral Village, a Louisiana Tribe Fights to Stay Put.” Natural Resources Defense Council (April 13, 2020) <https://www.nrdc.org/onearth/sea-level-rise-threatens-their-ancestral-village-louisiana-tribe-fights-stay-put>. (Last accessed on September 17, 2020).

^25^
Halle Parker. “For Louisiana's Coastal Tribes ‘Being at the End of the Earth is a Dangerous Place.’” The New Orleans Advocate Times Picayune (September 6 2021). <https://www.nola.com/news/environment/article_8751e728-0d8a-11ec-9fa1-cb44d2a508a1.html>. (Last accessed on October 2, 2021).

^26^
Julie Dermansky. “10 Days After Hurricane Ida, Historic Black Louisiana Town Contends with Scattered Coffins as Floodwaters Drain from Streets.” DeSmog (September 14, 2021). <https://www.desmog.com/2021/09/14/hurricane-ida-ironton-louisiana-scattered-coffins-floodwaters-environmental-justice/>. (Last accessed on October 2, 2021).

^27^
Christophe Béné, Christophe, Lyla Mehta, Gordon McGranahan, Terry Cannon, Jaideep Gupte, and Thomas Tanner. “Resilience as a Policy Narrative: Potential and Limits in the Context of Urban Planning.” *Climate and Development* 10 (2017): 116–133.

^28^
Terry Cannon and Detlef Müller-Mahn. “Vulnerability, Resilience, and Development Discourse in Context of Climate Change.” *Natural Hazards* 55 (2010): 621–635.

^29^
Simone Domingue. “Who Knows What Comes Tomorrow: A Study of Resilience Discourse, Practice, and Politics in a Post-Disaster Field.” *Environmental Sociology* 6 (2019): 19–30.

^30^
Richard Friend and Marcus Moench. “What is the Purpose of Urban Climate Resilience? Implications for Addressing Poverty and Vulnerability.” *Urban Climate* 6 (2013): 98–113.

^31^
Robert D. Bullard and Beverly Wright. *The Wrong Complexion for Protection*. (New York, New York University Press, 2012).

^32^
Bill M. Jesdale, Rachel Morello-Frosch, and Lara Cushing. “The Racial/Ethnic Distribution of Heat Risk–Related Land Cover in Relation to Residential Segregation.” *Environmental Health Perspectives* 121 (2013): 811–817.

^33^
Lori Peek. “America's Deathrap Schools.” New York Times (April 7, 2018). <https://www.nytimes.com/2018/04/07/opinion/sunday/americas-deathtrap-schools.html>. (Last accessed on February 27, 2021).

^34^
Linda Shi, Eric Chu, Isabelle Anguelovski, Alexander Aylett, Jessica Debats, Kian Goh, Todd Schenk, Karen C. Seto, David Dodman, Debra Roberts, J. Timmon Roberts, and Stacy VanDeever. “Roadmap Towards Justice in Urban Climate Adaptation Research.” *Nature Climate Change* 6 (2016): 131–137.

^35^
Kevin Loughran and James R. Elliott. “Residential Buyouts as Environmental Mobility: Examining Where Homeowners Move to Illuminate to Illuminate Social Inequalities in Climate Adaptation.” *Population Environment* 41 (2019): 52–70.

^36^
Kevin Loughran, James R. Elliott, and S. Wright Kennedy. “Urban Ecology in the Time of Climate Change: Houston, Flooding, and the Case of Federal Buyouts.” *Social Currents* 6 (2019): 121–140.

^37^
Earthea Nance and Jamila Johnson. “Barriers to Equity in Flood Mitigation Buyouts.” <http://works.bepress.com/nanceea/28/>. (Last accessed on February 27, 2021).

^38^
Sandy Kerr, John Colten, Kate Johnson, and Glen Wright. “Rights and Ownership in Sea Country: Implications of Marine Renewable Energy for Indigenous and Local Communities.” *Marine Policy* 52 (2015): 108–115.

^39^
Anthony M. Levenda, Ingrid Behrsin, and Francesca Disana. “Renewable Energy for Whom? A Global Systematic Review of the Environmental Justice Implications of Renewable Energy Technologies.” *Energy Research and Social Science* 71 (2021): 101837.

^40^
Tracey M. Osborne. “Carbon Forestry and Agrarian Change: Access and Land Control in a Mexican Rainforest.” *Journal of Peasant Studies* 38 (2011): 859–883.

^41^
Isabelle Anguelovski, Linda Shi, Eric Chu, Daniel Gallagher, Kian Goh, Zachary Lamb, Kara Reeve, and Hannah Teicher. “Equity Impacts of Urban Land Use Planning for Climate Adaptation: Critical Perspectives from the Global North and South.” *Journal of Planning Education and Research* 36 (2016): 333–348.

^42^
Kate D. Derickson. “The Racial Politics of Neoliberal Regulation in Post-Katrina Mississippi.” *Annals of the Association of American Geographers* 104 (2014): 889–902.

^43^
Dean Hardy, Richard A. Milligan, and Nic Heynen. “Racial Coastal Formation: The Environmental Injustice of Colorblind Adaptation Planning for Sea-Level Rise.” *Geoforum* 87 (2017): 62–72.

^44^
Jill L. Harrison. “Bureaucrats' Tacit Understandings and Social Policy Implementation: Unpacking the Deviation of Agency Environmental Justice Programs from EJ Movement Priorities.” *Social Problems* 63 (2016): 534–553.

^45^
Dayna N. Scott. “‘We Are the Monitors Now’: Experiential Knowledge, Transcorporeality and Environmental Justice.” *Social and Legal Studies* 25 (2016): 261–287.

^46^
Gwen Ottinger. *Refining Expertise: How Responsible Engineers Subvert Environmental Justice Challenges*. (New York, New York University Press, 2013).

^47^
Jill Harrison. *From the Inside Out: The Fight for Environmental Justice within Government Agencies*. (Cambridge, MIT Press, 2019).

^48^
Tristan Baurick. “Welcome to ‘Cancer Alley’ Where Toxic Pollution is About to Get Worse.” ProPublica (October 30, 2019) <https://www.propublica.org/article/welcome-to-cancer-alley-where-toxic-air-is-about-to-get-worse>. (Last accessed on October 2, 2021).

^49^
Sam Karlin. “Hurricane Ida Brings ‘Havoc’ to Huge Swaths of Louisiana, but Levees Prevent Mass Casualties.” New Orleans Advocate Times-Picayune (August 30, 2021). <https://www.nola.com/news/hurricane/article_a0a90bc2-09d2-11ec-a5fe-0b351e97fce1.html>. (Last accessed on October 2, 2021).

^50^
Christine Baniewicz. “Coastal Louisiana Tribes Team Up with Biologist to Protect Sacred Sites.” Southerly (September 2, 2020). <https://southerlymag.org/2020/09/02/coastal-louisiana-tribes-team-up-with-biologist-to-protect-sacred-sites-from-rising-seas/?fbclid=IwAR3vXnOi9sHD3giR0fG_4Torx85YVOjHQH40FPZIb29zZ1Lm4KQLcq_Mnyw>. (Last accessed on October 30, 2020).

^51^
Jill Harrison. *Pesticide Drift and the Pursuit of Environmental Justice*. (Cambridge, MIT Press, 2011).

^52^
Norman J. Vig and Michael E. Kraft. *Environmental Policy: New Directions for the 21st Century, 8th ed*. (Thousand Oaks, CQ Press, 2013).

^53^
Luzilda Carrillo Arciniega. “The Zero-Sum Game of White Supremacy.” Anthropology News (2017). DOI: 10.1111/AN.529.

^54^
Heather McGhee. *The Sum of Us: What Racism Costs Everyone and How We Can Prosper Together*. (New York, One World, 2021).

^55^
David Pellow. “Toward a Critical Environmental Justice Studies: Black Lives Matter as an Environmental Justice Challenge.” *Du Bois Review: Social Science Research on Race* 13 (2016): 221–236.

^56^
Colette Pichon-Battle, TED Talk <https://www.ted.com/speakers/colette_pichon_battle>. (Last accessed on September 3, 2021).

^57^
Colette Pichon-Battle, Gulf Coast Center for Law and Policy, State of the Coast Conference Keynote Address, June 2, 2021.

